# ALS skeletal muscle shows enhanced TGF-β signaling, fibrosis and induction of fibro/adipogenic progenitor markers

**DOI:** 10.1371/journal.pone.0177649

**Published:** 2017-05-16

**Authors:** David Gonzalez, Osvaldo Contreras, Daniela L. Rebolledo, Juan Pablo Espinoza, Brigitte van Zundert, Enrique Brandan

**Affiliations:** 1 Centro de Envejecimiento y Regeneración, CARE Chile UC y Departamento de Biología Celular y Molecular, Facultad de Ciencias Biológicas, Pontificia Universidad Católica de Chile, Santiago, Chile; 2 Centro de Investigaciones Biomédicas, Facultad de Ciencias Biológicas y Facultad de Medicina, Universidad Andres Bello, Santiago, Chile; University of Minnesota Medical Center, UNITED STATES

## Abstract

Amyotrophic lateral sclerosis (ALS) is a fatal neurodegenerative disease in which upper and lower motoneurons degenerate leading to muscle wasting, paralysis and eventually death from respiratory failure. Several studies indicate that skeletal muscle contributes to disease progression; however the molecular mechanisms remain elusive. Fibrosis is a common feature in skeletal muscle under chronic damage conditions such as those caused by muscular dystrophies or denervation. However, the exact mechanisms of fibrosis induction and the cellular bases of this pathological response are unknown. We show that extracellular matrix (ECM) components are augmented in skeletal muscles of symptomatic hSOD1^G93A^ mice, a widely used murine model of ALS. These mice also show increased TGF-β1 mRNA levels, total Smad3 protein levels and p-Smad3 positive nuclei. Furthermore, platelet-derived growth factor receptor-α (PDGFRα), Tcf4 and α-smooth muscle actin (α-SMA) levels are augmented in the skeletal muscle of symptomatic hSOD1^G93A^ mice. Additionally, the fibro/adipogenic progenitors (FAPs), which are the main producers of ECM constituents, are also increased in these pathogenic conditions. Therefore, FAPs and ECM components are more abundant in symptomatic stages of the disease than in pre-symptomatic stages. We present evidence that fibrosis observed in skeletal muscle of symptomatic hSOD1^G93A^ mice is accompanied with an induction of TGF-β signaling, and also that FAPs might be involved in triggering a fibrotic response. Co-localization of p-Smad3 positive cells together with PDGFRα was observed in the interstitial cells of skeletal muscles from symptomatic hSOD1^G93A^ mice. Finally, the targeting of pro-fibrotic factors such as TGF-β, CTGF/CCN2 and platelet-derived growth factor (PDGF) signaling pathway might be a suitable therapeutic approach to improve muscle function in several degenerative diseases.

## Introduction

Amyotrophic lateral sclerosis (ALS) is a neurodegenerative disorder characterized by progressive degeneration of upper and lower motoneurons. The loss of motoneurons leads to muscle atrophy, spasticity, paralysis and death from respiratory failure, usually within 3–5 years of diagnosis [1, 2]. Most ALS cases (~90%) are sporadic, whereas the remaining ~10% corresponds to familial cases (fALS). Mutations in the superoxide dismutase 1 gene (SOD1) account for 10–20% of all fALS cases. Recently, a hexanucleotide repeat expansion in the C9ORF72 gene was found to explain more than 50% of fALS cases [3, 4]. It has been shown that overexpression of the mutant human SOD1 gene in mice reproduces the ALS phenotype observed in humans [5]. This model was useful to identify pathological alterations in motoneurons such as mitochondrial dysfunction, glutamate excitotoxicity, hyper-excitability, axonal transport deficits and protein aggregation, among others [6]. However, the mechanism by which mutant SOD1 becomes toxic remains elusive.

ALS is partially considered a non-cell autonomous disease, and several studies have shown that non-neuronal cells can contribute to disease progression. For instance, astrocytes that express mutant SOD1, and more recently mutant TDP-43, selectively kill motoneurons by a non-cell-autonomous mechanism [7–11]. Other studies suggest that microglia have enhanced redox stress and induce motoneurons injury when they are incubated in presence of extracellularly added mutant SOD1^G93A^ [12, 13]. Nevertheless, whether skeletal muscle contributes to motoneuron degeneration and disease progression is not well established to date and the available evidence is still controversial. It has been shown that suppression of SOD1^G93A^ by gene transfer in skeletal muscle does not delay disease progression nor does improve muscle function [14], suggesting that skeletal muscle is not a primary target in ALS. However, other studies have shown that the skeletal muscle is in fact, a primary target of SOD1^G93A^ toxicity and can induce degeneration of motoneurons, contributing to disease onset [15, 16]. However, the molecular mechanisms by which skeletal muscle could be triggering neurodegeneration are not yet understood.

Fibrosis is a process that underlies several chronic disorders and involves the replacement of functional tissue by excessive non-functional connective tissue composed mainly by extracellular matrix (ECM), which affects normal cell physiology [17–19]. The fibrotic process in skeletal muscle has been well studied in the *mdx* mice, a murine model of Duchenne Muscular Dystrophy (DMD) [20–22]. It is known that several factors are involved in skeletal muscle fibrosis [23–25] and one of the best studied molecules is the transforming growth factor type-β (TGF-β), a central pro-fibrotic factor that is up-regulated in skeletal muscles of *mdx* mice [26–30]. The canonical TGF-β or Smad-dependent signaling pathway involves the direct phosphorylation of Smad2/3 by TGF-β receptor I kinase. Phosphorylated Smad2/3 binds to Smad4 and translocate into the nucleus [31, 32]. Several studies show elevated TGF-β1 levels in the serum and plasma from ALS patients [33, 34]. Intraperitoneal TGF-β2 injections improve motoneuron health and motor performance, however, they do not prevent motoneuron degeneration and mouse health deterioration [35]. A recent study shows strong evidence that TGF-β1 secreted by astrocytes from the spinal cord has a key role in the neuroprotective inflammatory response in ALS mice. However, excess of TGF-β1 from glia appears to have a negative role in neuroinflammation and blocking the TGF-β signaling pathway in a cell type-specific manner could be a therapeutic approach for neurodegenerative disorders [36]. It has been shown that TGF-β and Smad expression in skeletal muscle correlates with disease progression and could be a strong marker for ALS [37]. However, the role of TGF-β and ECM deposition in skeletal muscle from ALS mice models has not been studied.

Fibro/adipogenic progenitors (FAPs) are interstitial populations of mesenchymal cells that participate directly in fatty and fibrous connective tissue deposition in the skeletal muscle [38, 39]. These mesenchymal progenitors specifically express PDGFRα and Tcf4 [40, 41], and are increased in numbers in different models of chronic muscle damage [42–45]. Moreover, FAPs play a supporting role in healthy muscle regeneration [41, 46, 47].

In this work, we describe that elevated fibrosis correlates with enhanced TGF-β signaling in skeletal muscle of symptomatic hSOD1^G93A^ mice. We also found that several fibro/adipogenic progenitor (FAPs) markers are increased, and that they could be responsible for ECM deposition observed in the skeletal muscle from symptomatic hSOD1^G93A^ mice. Understanding the factors and cell types involved in exacerbated ECM synthesis and deposition is critical to further comprehend this devastating pathology.

## Materials and methods

### 2.1 Animals and tissue collection

All protocols were conducted in strict accordance and with the formal approval of the Animal Ethics Committee of the Pontificia Universidad Católica de Chile. The hemizygous transgenic mice carrying the mutant human SOD1^G93A^ (B6SJL-Tg (SOD1*G93A) 1Gur/J) gene were originally obtained from Jackson Laboratories (Bar Harbor, USA). Wild-type (B6SJL) and hSOD1^G93A^ age-matched male mice (60 and 120 days old) were anesthetized with 3.0% isoflurane gas in pure oxygen and sacrificed by cervical dislocation. The gastrocnemius muscle was then dissected and removed. Tissues for western blot analysis were rapidly frozen in liquid nitrogen. Tissue samples for cryosectioning were frozen in liquid nitrogen cooled-isopentane (Merck, Darmstadt, GE) and stored at -80°C until processing [27].

### 2.2 Histology and Sirius red staining

Gastrocnemius muscle cryosections (7μm) were placed onto glass slides. Hematoxylin and eosin staining was performed to assess muscle architecture and histology. Briefly, tissue sections were incubated for 10 minutes in formalin (10% v/v), then washed with water, incubated for 5 minutes with diluted hematoxylin (Merck, Darmstadt, GE; 25% v/v in H_2_O) and washed in H_2_O. Eosin was added for 30 seconds and then dehydration with ethanol was performed. Finally, Entellan was added to the slices [27]. Total collagen content was evaluated by staining with 1% Sirius red in picric acid [28]. Briefly, sections were fixed in 100% ethanol at 4°C for 30 minutes, washed with H_2_O, incubated in picric acid at 50°C for 1 hour, washed with H_2_O at least three times and incubated for 5 minutes with a solution of direct Red plus picric acid protected from light. Sections were then washed briefly with acetic acid 2% v/v, ethanol 50% v/v plus picric acid 50% v/v in ethanol. A final dehydration was performed and xylol was added. Sections were imaged using bright field microscopy on a Nikon Eclipse E600 [48].

### 2.3 Immunofluorescence microscopy

For skeletal muscle immunofluorescence, cryosections (7μm) were fixed in 4% paraformaldehyde (Merck, Darmstadt, GE), blocked for 1 hour in blocking buffer (1% BSA, BM-0150, Winkler, Santiago, CL), 1% gelatin from cold water fish skin (G7765, Sigma, St. Louis, MO, USA), 0,01% Triton X-100 (X100-1L, Sigma, MO, St. Louis, USA) in PBS, and incubated overnight at 4°C with the following antibodies: anti-fibronectin (Sigma-Aldrich, St. Louis, MO, USA), anti-collagen-I (Abcam, Cambridge, UK), anti-p-Smad3 (Cell Signaling, Danvers, MA, USA), anti-Tcf4 (Cell Signaling, Danvers, MA, USA), anti-PDGFRα (R&D Systems, Minneapolis, MN, USA) anti-laminin (Sigma-Aldrich, St. Louis, MI, USA). The corresponding Alexa Fluor 568 or 488-conjugated anti IgGs (Invitrogen, Carlsbad, CA, USA) were used as secondary antibodies. For nuclear staining, sections were incubated with 1 μg/mL Hoechst 33258 in PBS for 10 minutes [42]. Slices were then washed in water and mounted in fluorescent mounting medium (DAKO, Santa Clara, CA, USA). Sections were visualized on a Nikon Eclipse E600 epifluorescence microscope or a Nikon Eclipse C2 si confocal spectral microscope using NIS-Elements software v4.20, 32 bit.

### 2.4 Immunoblot analysis

Muscles were homogenized in 10 volumes of Tris-EDTA buffer pH7.4 with 1mM phenylmethylsulfonyl fluoride (PMSF). Then, one volume of buffer containing 2% glycerol, 4% SDS and 0.125 M Tris pH 6.8 were added to the homogenates as described previously [27]. Protein concentration was determined in aliquots of muscle extracts using the Bicinchoninic Acid Protein Assay kit (Pierce, Rockford, IL, USA) with BSA as the standard. Aliquots (50μg) were subjected to sodium dodecyl sulphate gel electrophoresis (SDS-PAGE) in 9% polyacrylamide gels, electrophoretically transferred onto PVDF membranes (Millipore, Billerica, MA, USA) and probed with the following antibodies at 4°C overnight: anti-fibronectin (Sigma-Aldrich, St. Louis, MO, USA), anti-collagen-III (Rockland, Limerick, PA, USA), anti-Smad3 (Cell Signaling, Danvers, MA, USA), anti-PDGFRα R&D Systems, Minneapolis, MN, USA (), anti-Tcf4 (Cell Signaling, Danvers, MA, USA), α-SMA (Sigma-Aldrich, St. Louis, MO, USA), anti-TGF-β(1, 2, 3) (R&D Systems, USA), anti-CTGF (Santa Cruz, USA) and anti-GAPDH (Millipore, Billerica, MA, USA). Primary antibodies were detected with horseradish-peroxidase-conjugated secondary antibodies after 1h incubation at room temperature. All immunoreactions were visualized by enhanced chemiluminescence (Pierce, Rockford, IL, USA). Densitometric analysis and quantification were performed using the ImageJ software (NIH, USA).

### 2.5 RNA isolation, reverse transcription and quantitative real-time PCR

Total RNA was isolated from gastrocnemius muscle using TRIzol (Invitrogen, Carlsbad, CA, USA) according to the manufacturer’s instructions. Total RNA (2μg) was reverse-transcribed into cDNA using random hexamers and M-MLV reverse transcriptase (Invitrogen, Carlsbad, CA, USA). TaqMan quantitative real-time PCR reactions were performed in duplicate on an Eco Real-Time PCR System (Illumina, San Diego, CA, USA) using predesigned primer sets for mouse transforming growth factor-β1 gene (TGF- β1; Mm01178820_m1) and the housekeeping gene GAPDH Mm99999915_g1; TaqMan Assays-on-Demand, Applied Biosystems, Foster City, CA, USA) or using primer set for rat-mouse PDGFRα (F.P: 5’ CGACTCCAGATGGGAGTTCCC 3’, R.P: 5’ TGCCATCCACTTCACAGGCA 3’), mouse Zfand5 (F.P: 5’ CAGAGCGTCAGTCGTCGGGATC 3’, R.P: 5’ GGTAGGACTGTTGGAACCACTAGC 3’), mouse Schip1 (F.P: 5’ GCACAATGGCAACGTGGTGGTAGC 3’, R.P: 5’ CCGTCTTACTGTCATCTGCATCGCTG 3’) and 18S (F.P: 5’ TGACGGAAGGGCACCACCAG 3’, R.P: 5’ CACCACCACCCACGGAATCG 3’). (mRNA expression was quantified with the comparative dCt method (2-ddCt), using GAPDH or 18S as reference genes. The mRNA levels were expressed relative to the mean expression in the wild-type mice.

### 2.6 Determination of fiber diameter

Fresh-frozen gastrocnemius muscles were sectioned and cryosections (7 μm) were placed on glass slides, fixed in 4% paraformaldehyde (Merck, Darmstadt, GE) and incubated with Alexa Fluor 594 conjugated-wheat germ agglutinin (WGA) (Thermo Fisher, Waltham, MA, USA) in PBS for 2 hours. Fiber size was calculated using the ImageJ software (NIH, USA) on reconstructed images of each gastrocnemius muscle. Fibers were manually selected and the minimal Feret diameter of each fiber was computed by the software [48].

### 2.7 Statistical analyses

The statistical significance of the differences between the means of the experimental groups was evaluated using one-way ANOVA or two-way ANOVA when appropriate, with a *post hoc* Bonferroni multiple comparison test. A difference was considered statistically significant with a *P*-value < 0.05. Data analyses and statistical analysis for all experiments were performed using Prism 5 software (GraphPad Software, CA, USA).

## Results

### 3.1 Symptomatic ALS skeletal muscle shows significant atrophy and increased fibrosis

**Fig 1:**
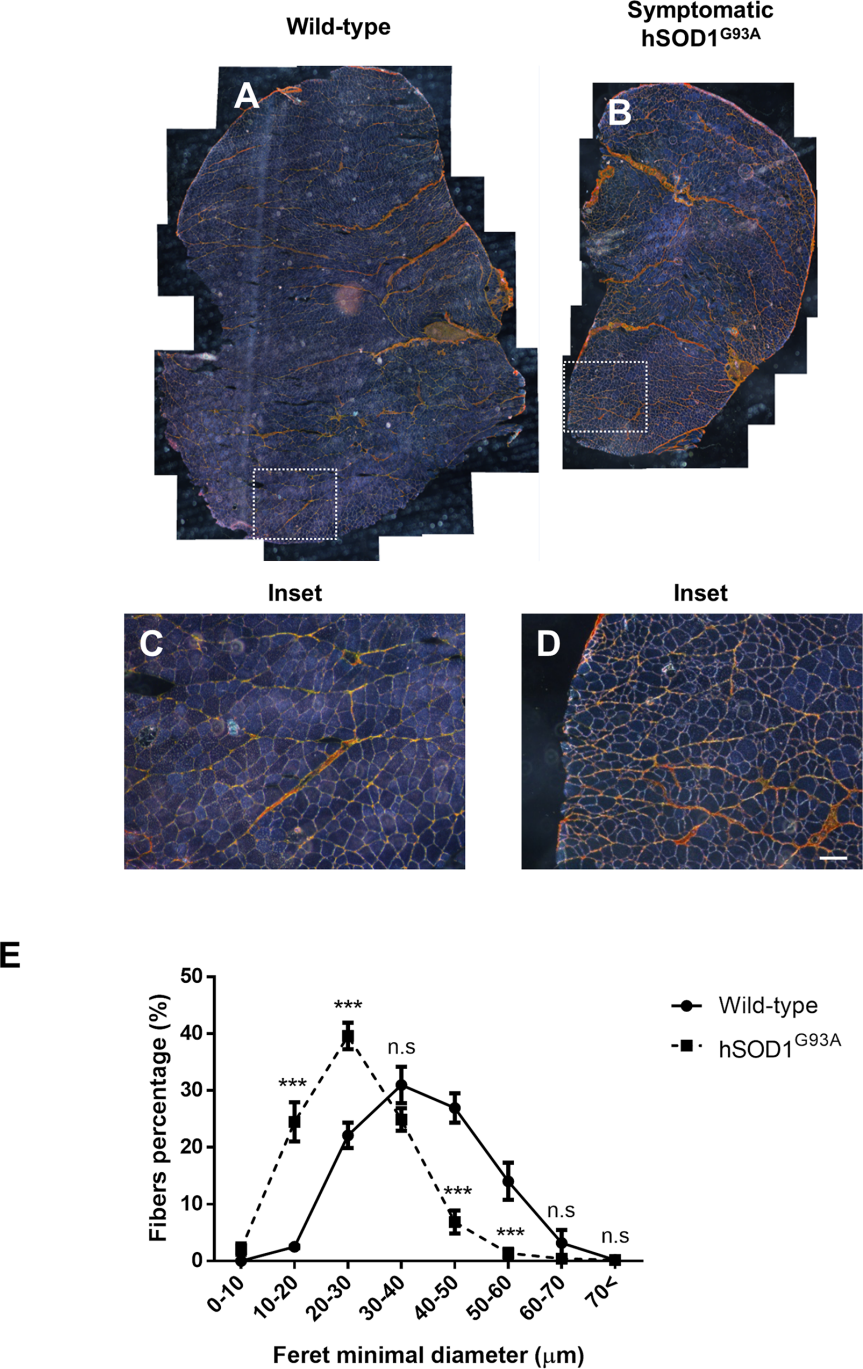
Muscle architecture is altered in gastrocnemius muscle from symptomatic hSOD1^G93A^ mice. **(A-D)** Sirius red staining of gastrocnemius muscle from wild-type (120 days old) and symptomatic (120 days old) hSOD1^G93A^ age-matched mice seen under polarized light in cross-sections. Bar corresponds to 100 μm. **(E)** Fiber diameter in wild-type (120 days old; black circles) and symptomatic hSOD1^G93A^ (120 days old; black squares) mice, values correspond to the mean ± SEM of three animals for each experimental condition. Two-way ANOVA, *** p<0.001, n.s: not significant.

**Fig 2:**
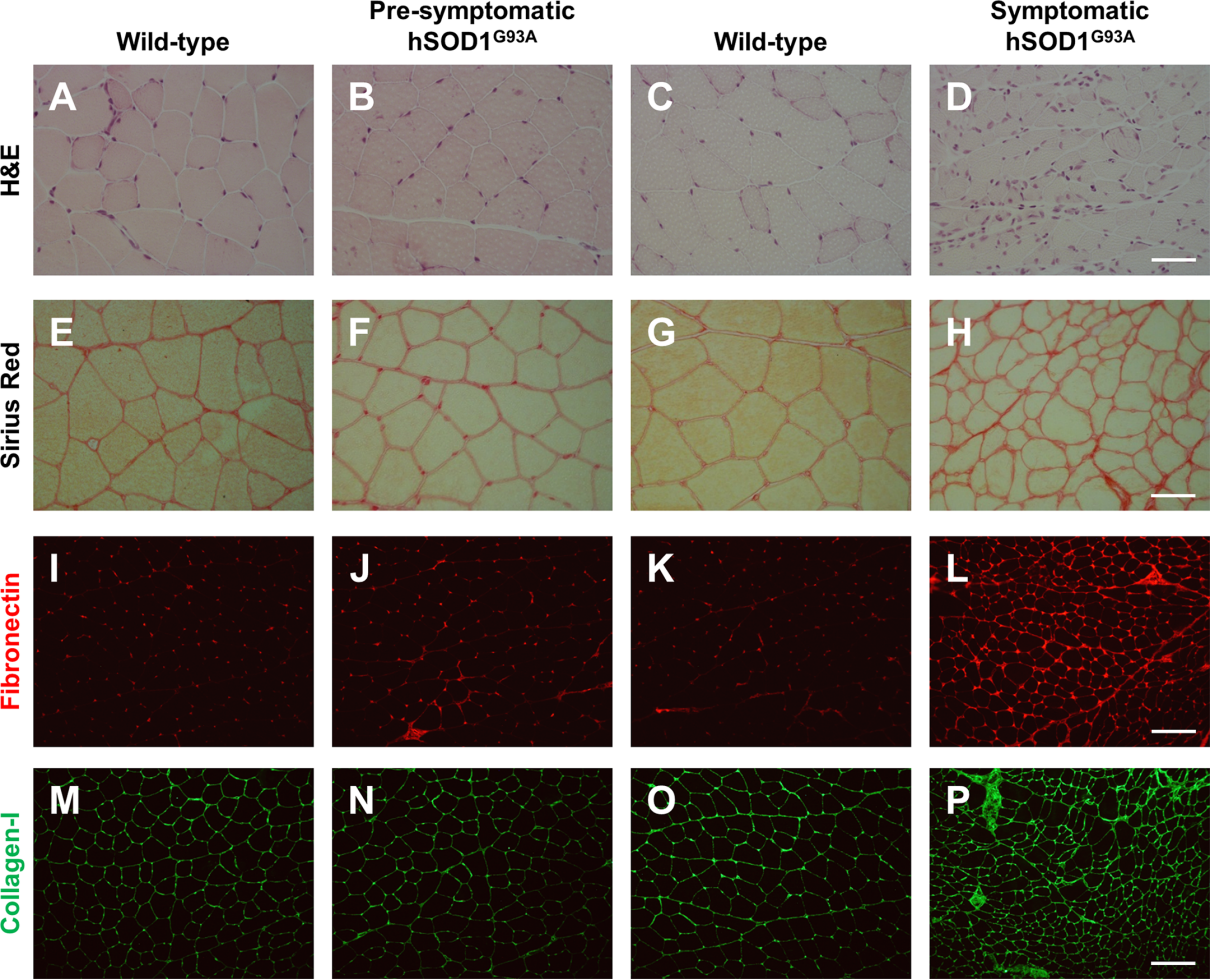
Extracellular matrix (ECM) components deposition in gastrocnemius muscle from symptomatic hSOD1^G93A^ mice. **(A-D)** H&E staining. **(E-H)** Sirius red staining. **(I-L)** fibronectin (red) and **(M-P)** collagen-I (green) were detected by indirect immunofluorescence in cross-sections of gastrocnemius muscle from wild-type (60 days old), hSOD1^G93A^ pre-symptomatic (60 days old), wild-type (120 days old), and hSOD1^G93A^ symptomatic (120 days old) age-matched mice. Bar corresponds to 50 μm. Representative images of three mice per condition.

Skeletal muscle sections obtained from hSOD1^G93A^ mice (ALS animal model) gastrocnemius muscle during symptomatic stages (120 days old), and from age matched wild type littermates, were analyzed by sirius red staining, a technique used to estimate total collagen. The samples were observed under polarized light. Fig 1A and 1C shows the normal architecture of wild-type mice skeletal muscle. In contrast, skeletal muscles from symptomatic hSOD1^G93A^ mice show substantial atrophy with high variation in fiber size and accumulation of collagen evidencing a fibrotic process (Fig 1B and 1D). The atrophy of myofibers was further determined by analyzing the frequency of muscle fibers size. Fig 1E shows the analysis of gastrocnemius myofibers diameter indicating a significant decrease in the Feret minimal diameter in symptomatic hSOD1^G93A^ mice compared to wild type littermates. This result suggests significant skeletal muscle atrophy in symptomatic hSOD1^G93A^ mice. Gastrocnemius muscle extracted from symptomatic (120 days old) and pre-symptomatic (60 days old) hSOD1^G93A^ mice as well as age matched wild type littermates were analyzed by hematoxylin and eosin staining. Fig 2A–2D shows the normal architecture of skeletal muscle from pre-symptomatic hSOD1^G93A^ mice compared to wild type mice. We observed fibers of homogeneous size with peripherally located nuclei that most likely correspond to fibers and interstitial cells. In contrast, skeletal muscle from symptomatic hSOD1^G93A^ mice show a substantial increase in interstitial nuclei that are mainly located surrounding packages of muscle fibers at the perimysium and around individual fibers in the endomysium. We also observed thickening of perimysium and endomysium, suggesting an increase in ECM components. To evaluate this, skeletal muscle sections were stained with sirius red. Fig 2E–2H indicates that symptomatic hSOD1^G93A^ gastrocnemius muscle shows increased sirius red staining and decreased fiber size.

**Fig 3:**
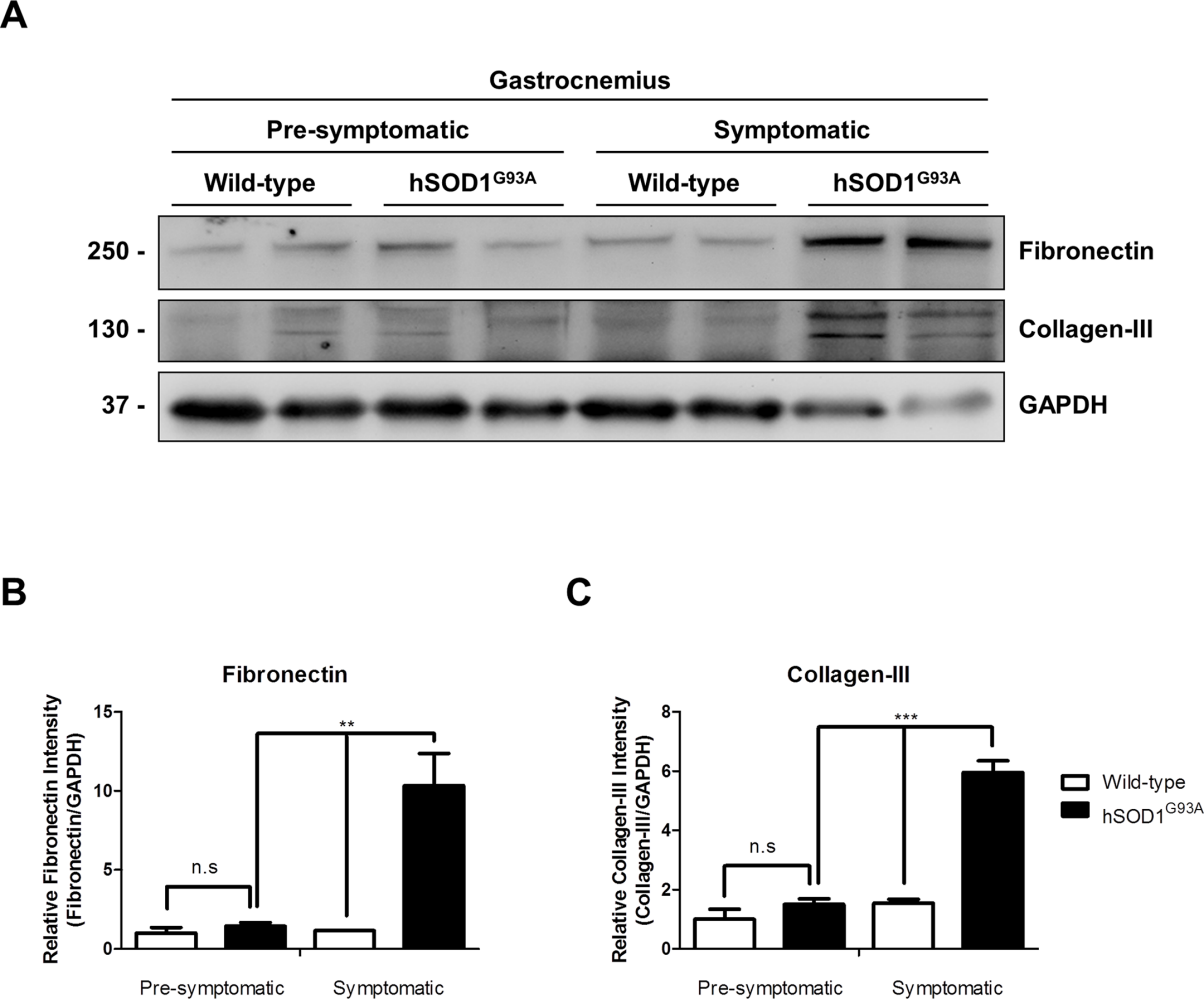
Extracellular matrix (ECM) components are augmented in gastrocnemius muscle from symptomatic hSOD1^G93A^ mice. **(A)** Fibronectin and collagen-III were detected by western-blot in protein extracts from wild-type mice (60 days old), pre-symptomatic (60 days old) hSOD1^G93A^ age-matched mice, wild-type (120 days old), and symptomatic (120 days old) hSOD1^G93A^ age matched mice. GAPDH protein levels are shown as loading control. **(B-C)** Protein levels of fibronectin and collagen-III were quantified using densitometric analysis. Values correspond to the mean ± SEM of four animals for each experimental condition. One-way ANOVA, ** p<0.05; *** p<0.001, n.s: not significant.

In order to confirm the increased fibrosis observed in hSOD1^G93A^ mice suggested by sirius staining (Fig 2E–2H), we immunostained muscle sections with specific antibodies against the ECM proteins fibronectin and collagen type I. Fig 2I–2P shows that these two fibrotic proteins are more abundant in muscle from symptomatic hSOD1^G93A^ mice compared to age matched wild type and pre-symptomatic hSOD1^G93A^ mice. The increased staining was observed either at the perimysium or endomysium. To further evaluate and quantify the increment of ECM proteins, skeletal muscle extracts obtained from each experimental condition were analyzed by western blot. Fig 3A shows a representative western blot for fibronectin and collagen type III, two ECM constituents. An increase in the amount of these two fibrotic proteins was observed only in the muscle samples obtained from symptomatic hSOD1^G93A^ mice compared to age matched wild type animals. No increase in these proteins was observed in pre-symptomatic stages of hSOD1^G93A^ mice. Fig 3B and 3C shows a quantitative analysis of the significant increase of these two proteins from symptomatic hSOD1^G93A^ compared to pre-symptomatic mice. Taken together, these results show that in the skeletal muscle of symptomatic hSOD1^G93A^ mice there is increased atrophy. This becomes evident by a shift to the left in the distribution curve of Feret minimal diameter of the myofibers, together with a significant increase in fibrosis. These changes were not observed in pre-symptomatic hSOD1^G93A^ mice.

### 3.2 TGF-β signaling pathway is induced in gastrocnemius muscle from symptomatic hSOD1^G93A^ mice

**Fig 4:**
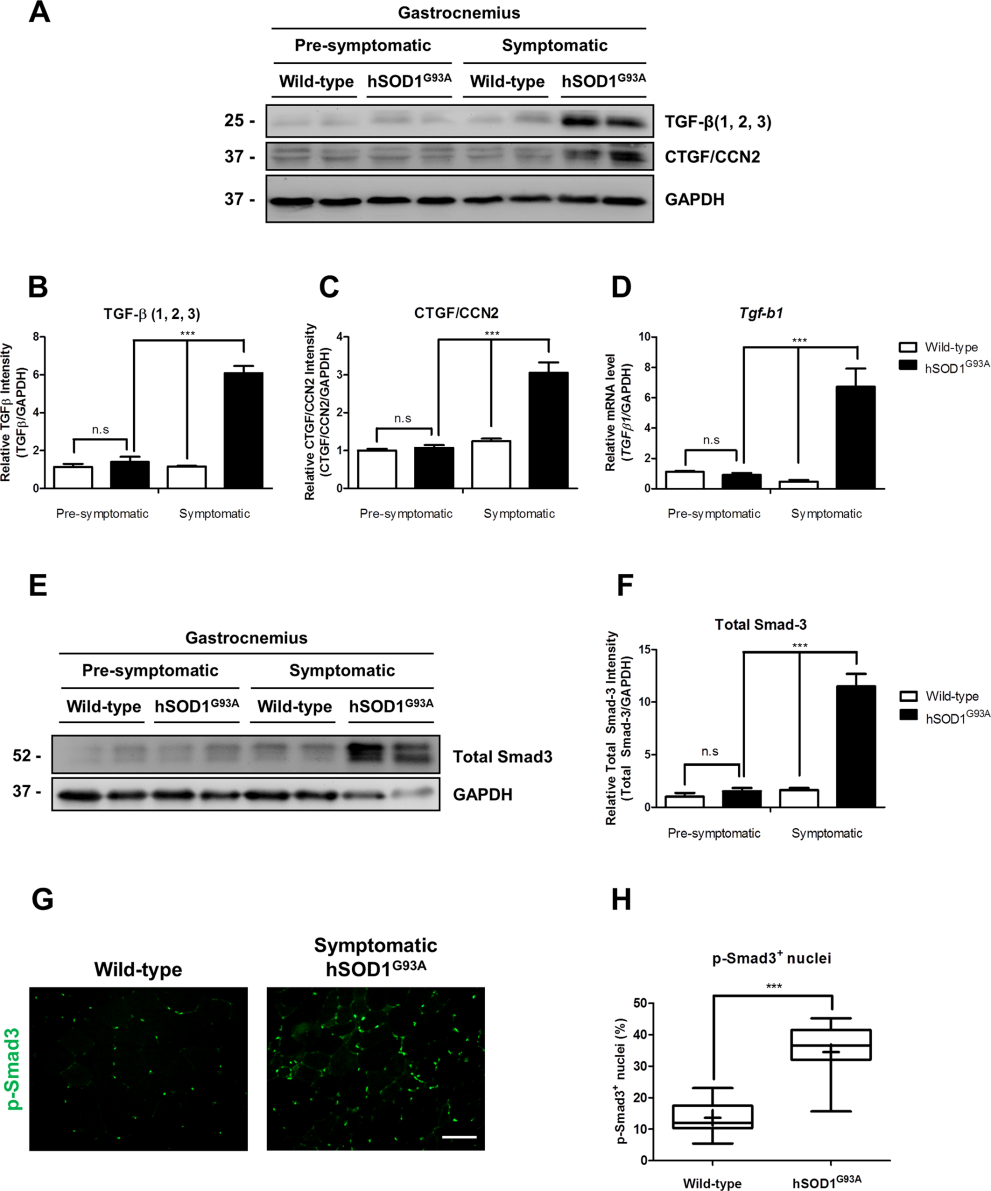
TGF-β signaling pathway is induced in gastrocnemius muscle from symptomatic hSOD1^G93A^ mice. **(A)** TGF-β (1, 2, 3), CTGF/CCN2 was detected by western-blot in protein extracts under non-reducing condition from wild-type (60 days old), pre-symptomatic (60 days old) hSOD1^G93A^ age-matched mice, wild-type (120 days old) and symptomatic (120 days old) hSOD1^G93A^ age-matched mice. GAPDH protein levels are shown as loading control. **(B-C)** Protein levels of TGF-β (1, 2, 3) and CTGF/CCN2 were quantified using densitometric analysis. Values correspond to the mean ± SEM of four animals for each experimental condition. One-way ANOVA, *** p<0.001. **(D)** *Tgf-b1* expression levels were analyzed by quantitative PCR comparing wild-type (60 days old), pre-symptomatic (60 days old) hSOD1^G93A^ age-matched mice, wild-type (120 days old) and symptomatic (120 days old) hSOD1^G93A^ age-matched mice. Values correspond to the mean ± SEM of four animals for each experimental condition. One-way ANOVA, *** p<0.001. **(E)** Total Smad3 was detected by western-blot in protein extracts from wild-type (60 days old), pre-symptomatic (60 days old) hSOD1^G93A^ age matched mice, wild-type (120 days old), and symptomatic (120 days old) hSOD1^G93A^ age-matched mice. GAPDH protein levels are shown as loading control. **(F)** Protein levels of total Smad3 were quantified using densitometric analysis. Values correspond to the mean ± SEM of four animals for each experimental condition. One-way ANOVA, *** p<0.001. **(F)** p-Smad3 (green) was detected by indirect immunofluorescence in cross-sections of gastrocnemius muscle from wild-type (120 days old) and hSOD1^G93A^ symptomatic (120 days old) age-matched mice, bar corresponds to 50 μm. **(G)** p-Smad3-positive nuclei were quantified in gastrocnemius muscle from wild-type (120 days old) and symptomatic (120 days old) hSOD1^G93A^ age-matched mice, values correspond to the mean ± SEM of three animals for each experimental condition. One-way ANOVA, *** p<0.001, n.s: not significant.

Since fibrosis was clearly augmented in symptomatic hSOD1^G93A^ mice, we decided to evaluate the state of TGF-β signaling pathway, a well characterized pro-fibrotic pathway under these experimental conditions. Fig 4A shows a representative western blot for the three isoforms of TGF-β and CTGF/CCN2, a pro-fibrotic factor acting downstream TGF-β signaling pathway. We observed an increase of these two pro-fibrotic factors only in symptomatic hSOD1^G93A^ mice. Quantitative analysis showed a 6-fold induction of TGF-β (1, 2, 3) and 3-fold induction of CTGF/CCN2 in symptomatic hSOD1^G93A^ mice compared to pre-symptomatic animals (Figs 4B and 1C). Fig 4D shows a significant increase in TGF-β1 mRNA transcripts in skeletal muscle obtained from hSOD1^G93A^ mice; in contrast, there was no significant difference in TGF-β1 mRNA levels in the pre-symptomatic stage. Next, we showed that there was no increase in total Smad3 in pre-symptomatic hSOD1^G93A^ (Fig 4E) compared to the age-matched wild type. In contrast, we found a significant increase in Smad3 in skeletal muscle from hSOD1^G93A^ mice compared to pre-symptomatic animals. Quantitative analyses indicate that there was a 10-fold increase in Smad3 in symptomatic hSOD1^G93A^ mice (Fig 4F). To evaluate if TGF-β signaling pathway was active, we determined the amount of phosphorylated Smad3 (p-Smad3) in skeletal muscle from symptomatic hSOD1^G93A^ and age-matched wild type mice by indirect immunofluorescence. Fig 4G shows a significant increase of nuclei positive for p-Smad3 in symptomatic hSOD1^G93A^ mice. Quantitative analysis shows that 36% of total nuclei were positive for p-Smad3 in symptomatic hSOD1^G93A^ mice compared to 14% in wild-type mice. These results suggest that TGF-β signaling pathway is activated in skeletal muscle of symptomatic hSOD1^G93A^ mice.

### 3.3 FAPs are augmented in symptomatic hSOD1^G93A^ skeletal muscle

**Fig 5:**
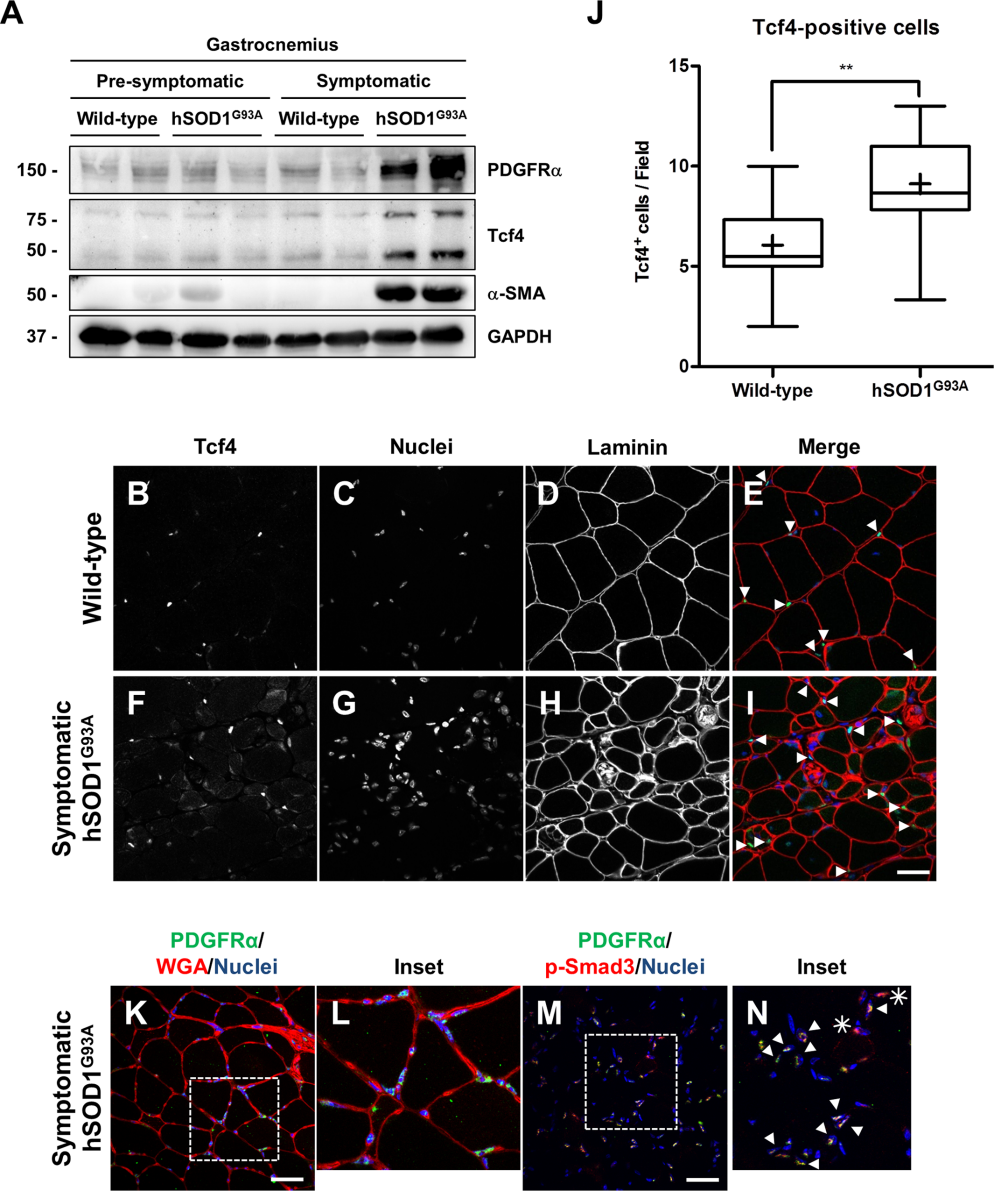
FAPs markers are increased in gastrocnemius muscle from symptomatic hSOD1^G93A^ mice. **(A)** PDGFRα, Tcf4 and α-SMA were detected by western-blot in protein extracts from wild-type (60 days old), pre-symptomatic (60 days old) hSOD1^G93A^ age-matched mice, wild-type (120 days old), and symptomatic (120 days old) hSOD1^G93A^ age-matched mice. GAPDH protein levels are shown as loading control. **(B-I)** Tcf4 (white and green) and laminin (white and red) were detected by indirect immunofluorescence in cross-sections of gastrocnemius muscle from wild-type (120 days old) and hSOD1^G93A^ symptomatic (120 days old) age-matched mice, bar corresponds to 50 μm, nuclei were stained with Hoechst. Arrows show Tcf4-positive nuclei. **(J)** Tcf4-positive cells were quantified in gastrocnemius muscle from wild-type (120 days old) and symptomatic (120 days old) hSOD1^G93A^ age-matched mice, values correspond to the mean ± SEM of three animals for each experimental condition. One-way ANOVA, ** p<0.005. **(K-L)** PDGFRα (green) and WGA (red) **(M-N)** PDGFRα and p-Smad3 (red) were detected by indirect immunofluorescence in cross-sections of gastrocnemius muscle from wild-type (120 days old) and hSOD1^G93A^ symptomatic (120 days old) age-matched mice, bar corresponds to 50 μm, nuclei were stained with Hoechst. Arrows and asterisk show PDGFRα/p-Smad3 co-localization and PDGFRα^-^/p-Smad3^+^ cells, respectively.

We previously showed that the number of FAPs is augmented in models of increased fibrosis [42]. Therefore, we evaluated their presence by western blot analyses using markers of activated FAPs. Fig 5A shows that the levels of PDGFRα, Tcf4 (transcription factor 7-like 2, Tcf7L2-Genome Mouse Browser) and α-SMA found in gastrocnemius muscle are significantly increased in symptomatic compared to pre-symptomatic hSOD1^G93A^ mice. Fig 5B–5I shows an important increase of positive interstitial nuclei for Tcf4 in gastrocnemius muscle from symptomatic hSOD1^G93A^ compared to age-matched wild type animals. Fig 5J, shows a quantitative analysis of the increment in cells positive for Tcf4 from symptomatic hSOD1^G93A^ mice compared to age-matched wild type mice. We observed that PDGFRα positive-cells reside in the interstitial space between myofibers located at the endomysium and perimysium of muscles from symptomatic hSOD1^G93A^ mice (Fig 5K and 5L). Fig 5M and 5N shows that cells positive for PDGFRα co-localize with nuclei positive for p-Smad3 in immunostaining experiments. To test whether a PDGF dependent signaling pathway is activated in hSOD1^G93A^, we evaluated expression of two PDGF immediate early genes (IEG), *Schip1* and *Zfand5*. We found an increase in mRNAs of these two genes in skeletal muscle of symptomatic hSOD1^G93A^ mice (S1 Fig). These results clearly indicate that activated FAPs, likely responsible of the augmented fibrosis, are elevated in symptomatic hSOD1^G93A^ mice compared to wild type littermates.

## Discussion

We have shown that skeletal muscle of symptomatic hSOD1^G93A^ mice exhibits altered architecture, atrophy and increased interstitial space between myofibers. This pathological phenotype is accompanied by deposition of ECM molecules such as fibronectin and collagen I and III. The accumulation of these molecules indicates that a fibrotic process has been established in symptomatic stages and, therefore, functional tissue is being replaced by “non-functional” connective tissue.

Fibrosis is one of the key features in several disorders including muscular dystrophies such as DMD, where the absence of dystrophin leads to degeneration/regeneration cycles of the myofibers that will eventually be replaced by connective tissue and fat [22, 49]. The causes of fibrosis are not fully clear. However, mounting evidence shows that an inflammatory response is required to trigger the fibrotic process [50, 51]. We have also observed that the number of mononuclear cells residing in the interstitial space between the myofibers is increased. Recent work has shown that in hSOD1^G93A^ rats, an active inflammatory response is occurring near the neuromuscular junction in skeletal muscle during symptomatic and end-stages. This inflammatory response is accompanied with an increase in CD11-b positive and CD68 positive macrophages and inflammatory cytokines IL1-β and TNF-α [52], suggesting that an inflammatory response might be preceding the fibrotic process in the skeletal muscle from ALS models.

We found an induction of the TGF-β signaling pathway in skeletal muscle of symptomatic hSOD1^G93A^ mice that correlates with an increase in ECM molecules deposition. This data supports a recent study that proposes TGF-β1, 2 and 3 as a novel muscle biomarker for ALS [37]. It has been shown that intraperitoneal injections of TGF-β2 leads to transient improvement in motor performance without preventing motoneurons degeneration and disease progression [53]. One possible explanation is that, besides its neuroprotective role, TGF-β might also be deleterious in ALS. As reported recently, administration of SB-431542 (a strong inhibitor of the TGF-β type 1 receptor) delays ALS disease progression, indicating that the induction of TGF-β signaling has a detrimental effect [36].

Several studies have shown that TGF-β signaling pathway disturbances are associated with muscular dystrophies including Duchennes’s and Becker’s [54–56]. It has been shown that TGF-β signal inhibition using a TGF-β-neutralizing antibody or the angiotensin II type 1 receptor blocker losartan (an) reduces fibrosis levels and restores muscle architecture and function in two neuromuscular disease mouse models: the *mdx* mice and the fibrillin-1-deficient mice (Fbn1^C1039G/+^, Marfan Syndrome) [26]. We previously reported that TGF-β inhibition through angiotensin-1-7 administration reduces fibrosis and improves strength in dystrophic muscles of *mdx* mice [27]. The connective tissue growth factor (CTGF/CCN2) is a strong pro-fibrotic factor acting downstream of the TGF-β signaling pathway, and is found at highly elevated levels in several injured tissues such us skin, lung and liver [57–60]. In previous studies we demonstrated that CTGF over-expression induces a fibrotic response in healthy skeletal muscle [61] while CTGF inhibition improves the pathological phenotype and reduces fibrosis [48]. This evidence indicates that TGF-β, and other pro-fibrotic factors such as CTGF, play a crucial role in the fibrotic response observed in several myopathies. Therefore, taking into account that we have found an induction in TGF-β signaling, together with an increase in ECM molecules deposition in skeletal muscle of symptomatic hSOD1^G93A^ mice, we speculate that targeting TGF-β and other pro-fibrotic factors may be a suitable therapeutic approach to restore, at least partially, muscle performance in ALS patients.

We found that FAPs markers PDGFRα and Tcf4 were highly expressed in skeletal muscles of symptomatic hSOD1^G93A^ mice and we also observed an increase in the number of Tcf4^+^ and PDGFRα cells mainly in the interstitial space between the myofibers. We have previously demonstrated that the number of connective tissue cells expressing FAPs markers are increased in three different fibrotic models: *mdx* mice, BaCl_2_-induced chronic damage and sciatic nerve denervation [42], suggesting a role of these cells in fibrosis associated to skeletal muscle. It seems that FAPs cells are required for correct regeneration in skeletal muscle. However, under conditions where persistent damage occurs, these cells appear to play a rather deleterious role [62]. Current evidence suggests that mesenchymal progenitors that reside in connective tissue are probably the major contributors of ECM deposition in skeletal muscle, and that expression of fibrotic genes including different types of collagens, CTGF and α-SMA, take place in PDGFRα^+^ cells [42, 44]. We showed that PDGFRα^+^ cells co-localize with p-Smad3^+^ nuclei, suggesting that these cells are sensitive to TGF-β and might be responsible of ECM deposition in muscles of hSOD1^G93A^ mice. Thus, FAPs seems to be a principal actor in fibrosis observed in hSOD1^G93A^ mice and focusing on these cells might be a suitable therapeutic approach. The exact source of TGF-β to induce this response in FAPs in hSOD1^G93A^ mice has to be determined. It has been shown in *mdx* mice, that infiltrating macrophages express elevated level of TGF-β avoiding apoptosis of FAPs by a TNF mediated mechanism [63]. Moreover, we evaluated expression of *Schip1* and *Zfand5*, two specific IEG of the PDFG signaling pathway [64, 65]. We found an increase in expression of these two genes, suggesting an induction of PDGF signaling pathway in skeletal muscle of symptomatic hSOD1^G93A^ mice. The induction of PDGF signaling is probably contributing to the fibrotic process [47, 66] together with TGF-β signaling.

Another work shows that Adam12^+^ cells differentiate into α-SMA-expressing myofibroblasts under TGF-β stimulation [67]. This result suggests that FAPs cells might differentiate into α-SMA-expressing myofibroblasts to ultimately secrete a plethora of ECM molecules, thus, contributing to a fibrotic process under muscle insults.

We demonstrated that TGF-β-dependent signaling is enhanced in skeletal muscle in symptomatic hSOD1^G93A^ mice compared to pre-symptomatic animals; this was determined by elevated levels of TGF-β1 transcripts, elevated protein levels of TGF-β(1, 2, 3),total Smad3 protein and increased positive nuclei for phosphorylated Smad3. We and others [37] have found augmented levels of TGF-β (three isoforms) in symptomatic hSOD1G93A mice. The pro-fibrotic effect of TGF-β1 have been well-studied, however, controversial evidence regarding TGF-β3 exist. It has been shown that TGF-β3 is required for a suitable wound healing [68]. Other study showed that TGF-β3 is a poor inducer of pro-fibrotic molecules and differentiation to myofibroblast in vocal fold fibroblasts, when compared to TGF-β1 and TGF-β2. Furthermore, TGF-β3 administration ameliorates scar formation in vocal fold acute injury [69]. However, the role of TGF-β3 in ALS has not been evaluated to the date. Further studies are needed to understand the role of the three different isoforms of TGF-β in neurodegenerative diseases such as ALS.

As discussed above, FAPs are increased in skeletal muscle interstitial space in symptomatic hSOD1^G93A^ mice, and they are probably secreting fibrotic proteins. It is plausible to suggest that TGF-β dependent fibrotic signals could be inducing an increase in FAPs number and in the secretion of ECM constituents. CTGF is a potential candidate to play this pro-fibrotic signaling, since is expressed and secreted by muscle cells [23] and could be exerting its effects in the pro-fibrotic FAPs present at the interstitial space.

In conclusion, our work provides evidence showing a fibrotic phenotype in skeletal muscles of symptomatic hSOD1^G93A^ mice accompanied by the enhancement of the TGF-β signaling pathway. We have shown that FAPs cells are increased in this fibrotic context and could be involved in ECM deposition observed in symptomatic stages. Finally, targeting pro-fibrotic factors such as TGF-β might be a novel therapeutic approach to improve muscle function in several degenerative diseases.

## Supporting information

S1 Fig PDGF immediate early genes (IEG) *Zfand5* and *Schip1* are up-regulated in skeletal muscle from symptomatic hSOD1^G93A^ mice. RT-PCR analysis of **(A)** *Pdgfra*, **(B)** *Schip1* and **(C)** *Zfand5* expression in skeletal muscle from wild-type and symptomatic hSOD1^G93A^ age-matched mice, 18S is shown as housekeeping gene. **(D)** *Schip1* and **(E)** *Zfand5* expression levels were analyzed by quantitative PCR in skeletal muscle from wild-type and symptomatic hSOD1^G93A^ age-matched mice. Values correspond to the mean ± SEM of four animals for each experimental condition. One-way ANOVA, * p<0.05, n.s: not significant.
(TIF)

## Abbreviations

ALS: Amyotrophic lateral sclerosis
α-SMA: α-smooth muscle actin
CTGF/CCN-2: Connective Tissue Growth Factor
DMD: Duchenne muscular dystrophy
ECM: Extracellular matrix
fALS: Familial ALS
FAPs: Fibro/adipogenic progenitors
PDGF: Platelet-derived growth factor
PDGFRα: Platelet-derived growth factor receptor-α
SOD1: Superoxide dismutase 1
TGF-β: Transforming growth factor type-β
