# Supplementary figures and images for: ALS skeletal muscle shows enhanced TGF-β signaling, fibrosis and induction of fibro/adipogenic progenitor markers
Source: PLoS One. 2017 May 16;12(5):e0177649. doi: 10.1371/journal.pone.0177649 (PMC5433732; doi:10.1371/journal.pone.0177649)

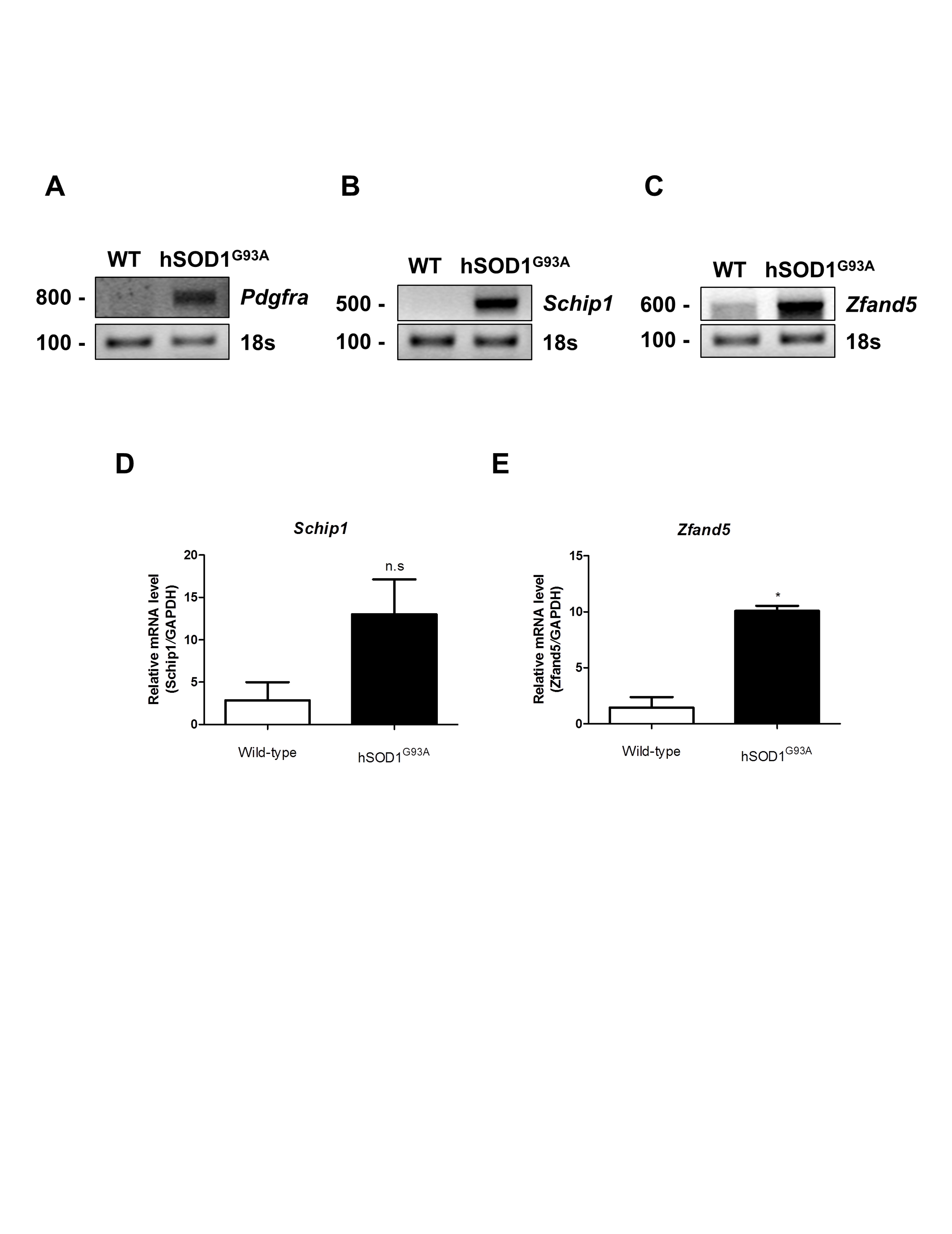

Supplement: S1 Fig — RT-PCR analysis of (A) Pdgfra, (B) Schip1 and (C) Zfand5 expression in skeletal muscle from wild-type and symptomatic hSOD1G93A age-matched mice, 18S is shown as housekeeping gene. (D) Schip1 and (E) Zfand5 expression levels were analyzed by quantitative PCR in skeletal muscle from wild-type and symptomatic hSOD1G93A age-matched mice. Values correspond to the mean ± SEM of four animals for each experimental condition. One-way ANOVA, * p<0.05, n.s: not significant. (TIF) [file pone.0177649.s001.tif]
